# Change in risk status of psychiatric patients admitted to Crisis and Home Treatment Team: an evaluation in the UK

**DOI:** 10.1192/j.eurpsy.2024.1188

**Published:** 2024-08-27

**Authors:** U. Raja, A. Misra, N. Kar

**Affiliations:** Black Country Healthcare NHS Foundation Trust, Wolverhampton, United Kingdom

## Abstract

**Introduction:**

The Crisis and Home Treatment Teams (CRHT) in psychiatry manages patients with risk to self and others in the community. The number of patents under CRHT who attempt or die of suicide is high in the UK (Hunt et al BJPsych Bull. 2016;40:172-4). The CRHT is an option to help support patients in managing their risk using various interventions and also aim to prevent admission to acute psychiatric wards where possible.

**Objectives:**

We intended to study the change in risk to self and others and the factors associated with it during the intervention from a CRHT taking care of adult patients in the West Midlands region of England.

**Methods:**

The study was conducted as a service evaluation of patients admitted under the CRHT. Data was collected from the case records, for 100 patients for whom details were available. Risk to self and others were checked, along with overall risk as red (highest risk), amber (intermediate risk) and green (low risk). Demographic and clinical information was collected and the data quality was checked.

**Results:**

There were 46 male and 54 female patients in the study, with mean age of 40.4 ± 12.4 and 40.2 ± 12.8 Years respectively (not significant). They were comparable in number of diagnoses (mean 1.2 each) and number of days (22.2 ± 13.1 v 20.2 ± 17.8) in CRHT respectively. There was no significant association of risk with gender (56.3% females and 44.2% of males), being on benefits or type of accommodation the service users live at. Similarly, there was no significant difference of risk of self-harm based on ethnicity; it was noted that 61.2% of patients of British White ethnicity had a risk of self on admission compared to 41.7% Black and ethnic minority patients. On admission, 89% of patients were categorised as red, amber 8% and 1% green; which changed to 18%, 2% and 77% respectively (missing data was not included, so percentages do not add up to 100%). The risk to self was present in 46% on admission and 18% on discharge (p<0.005); and in 14% this risk continued without change. The risk to others on admission was recorded in 12% which was at 1% on the point of discharge (p<0.05). Eight people had both risk to self and others. In 15 patients the risk continued to remain in red category, while in two patients it changed from amber to red.

**Conclusions:**

The risk levels for patients admitted under the CRHT improved. The majority with overall high risk changed to majority presenting as low risk on discharge. The percentage of patients portraying a risk to self and others also decreased from admission to discharge. Although there was considerable decrease in risk, a proportion of patients did not have any change, or even an increase in their risk, which highlights need for additional risk management strategy for these patients in CRHT.

**Disclosure of Interest:**

None Declared

